# Techniques for transcatheter recanalization of completely occluded vessels and pathways in patients with congenital heart disease

**DOI:** 10.4103/0974-2069.74044

**Published:** 2010

**Authors:** Larry A Latson, Athar M Qureshi

**Affiliations:** Center for Pediatric and Congenital Heart Disease, Children’s Hospital, Cleveland Clinic, Cleveland, Ohio, USA

**Keywords:** Occlusion, recanalization, transcatheter

## Abstract

Occlusions of major vessels in patients with congenital heart disease may occur due to a variety of factors. These occlusions are often felt to be best addressed surgically; however, we and others have been successful in recanalizing most of these vessels in the catheterization laboratory. Most of these patients will require multiple procedures in the catheterization laboratory to ensure vessel patency and to facilitate vessel growth. Physicians performing the procedure should have a thorough understanding of the anatomic considerations for the intended procedure and have access to a variety of devices and equipment to optimize the result of the procedure. In this article, we review some of the technical aspects that are vital for the success of the procedure.

## INTRODUCTION

Patients with significant congenital heart disease have a relatively high risk of developing occlusions of major veins, arteries or surgical pathways during their lifetime due to multiple factors. Surgical procedures, chronic intravenous lines, repeat cardiac catheterizations and periods of low cardiac output may all contribute to the development of vascular occlusions at many different sites. Acute or chronic occlusions of some vessels may have little clinical impact. However, successful recanalization in many instances can have significant short-term and long-term benefits. Surgical approaches to occlusions of large vessels have been relatively successful. Attempts to surgically recanalize smaller vessels or vessels with relatively slow flow in small children or in the more peripheral arterial system have frequently been disappointing. A small but growing experience with transcatheter recanalization of the occluded vessels in this difficult population is encouraging, but is limited. A few of the situations where transcatheter recanalization has been successful include superior vena caval (SVC) syndrome,[1] acute and chronic occlusion of pulmonary arteries.[2–6] completely occluded Blalock-Taussig (BT) shunts,[7–14] completely occluded venous pathways after atrial redirection procedures,[15,16] occluded pulmonary veins,[17] congenital or acquired atretic segments of the aorta[18,19] and acutely or chronically occluded ilio-femoral arteries[20] and veins.[21,22] In this article, we will review some of the general principles and approaches for attempting transcatheter vascular recanalization that we and others have found successful.

## PROCEDURAL PLANNING CONSIDERATIONS

Any attempt to recanalize an occluded major vessel has a significant risk for unintended vascular perforation and bleeding. We recommended that these procedures be carried out with general anesthesia. Complete immobility of the patient is more certain than with simple sedation. The procedures tend to be relatively long and may involve multiple steps. Control of the airway in an emergency is essential. Multiple sites of vascular access are often required. Biplane fluoroscopy is highly recommended as the intended pathway of recanalization may appear relatively straight in one view but not well aligned in the other complementary view.

Equipment needs for vascular recanalization procedures may vary somewhat. It is almost always important, however, to have a variety of guidewires, low-profile balloons and stents available. Hydrophilic guidewires are superior to standard guidewires in crossing totally occluded segments in adult patients with peripheral vascular disease,[23] and we have had a similar experience in our pediatric and adult congenital heart patients. Guidewires with a soft tip may be beneficial for manipulation in newly recanalized vessels, but stiff guidewires may be important for the positioning of angioplasty or stent balloons. Hydrophilic catheters and microcatheters may be useful in wire exchanges. Snare catheters may be extremely helpful if there is access to the distal side of an occlusion. The opened snare can serve as a target during attempted perforation of the occlusion. If a perforating guidewire tip can be snared, control of the guidewire tip tremendously enhances the ability to safely force a larger balloon or exchange catheter through the occlusion over the guidewire.

In general, we feel that it is safer to attempt recanalization of the vessels in low-pressure systems with surrounding scar tissue than in high-pressure systems in which there has been no preceding surgery or inflammation. We feel significantly less trepidation about attempting recanalization of an SVC or pulmonary artery after a Glenn shunt than we feel when considering primary treatment of a congenitally atretic segment of the aorta. Many vascular occlusions can occur very early after surgery. In these situations, extra care is needed to be certain that a penetrating guidewire has not perforated a suture line before advancing a catheter over the guidewire. Dilation or stenting of fresh suture lines once an occlusion has been traversed can be accomplished reasonably safely with relatively conservative balloon sizes.[24,25]

In some situations, a patient with an acute vascular occlusion may be critically ill. This is especially true if a BT shunt becomes occluded in a patient with limited other sources of pulmonary blood flow. In critically ill patients, it may be best to establish the patient on extracorporeal membrane oxygenator (ECMO) prior to attempting catheter intervention. Intervention on a BT shunt through the side port of an arterial cannula in an ECMO circuit has even been reported.[10] Availability of an experienced assistant may significantly shorten the procedure and may be essential for manipulating the accessory catheters, snares, etc.

## IDENTIFY THE TARGET

**Figure 1:**
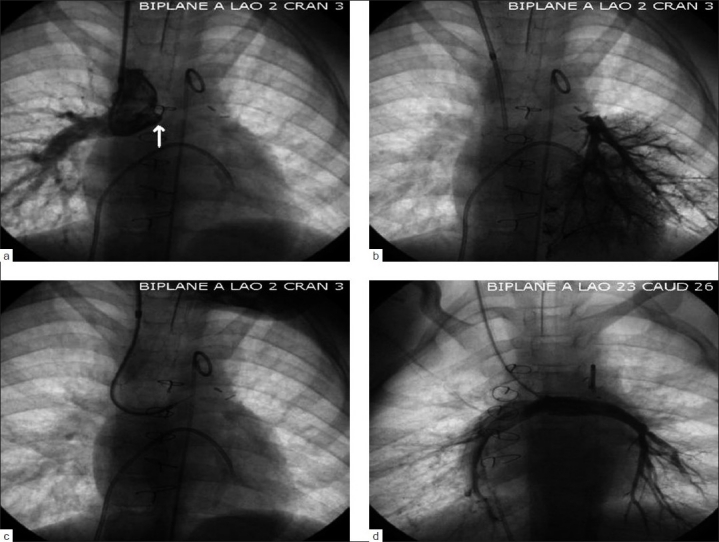
Angiograms in a 7-year-old girl with long-standing neo-left pulmonary artery occlusion (a) after an intial Norwood procedure and then subsequent bidirectional Glenn operation. Note the dimple (arrow) that helps direct the guidewire into the occluded vessel lumen. The distal left pulmonary artery is visualized with a left lower pulmonary vein wedge angiogram (b). The occluded segment was crossed using a guide and glide system and the stiff end of a 0.018" hydrophilic guidewire (c). Two Genesis XD 1910 stents (Cordis, Warren, NJ) were placed in series (mounted on 10-mm balloons) to establish vessel patency (d)

The presence of a funnel-shaped stump or a “dimple” in the area of occlusion helps to direct the catheter tip and ensure that an exploratory guidewire is directed toward a true lumen [Figure 1a]. Whenever possible, the target vessel past the occlusion should be visualized by placing a catheter in the distal segment or by roadmap images. Situations that may allow placement of a target catheter include discontinuous pulmonary arteries with shunts to both the right and the left pulmonary arteries, systemic venous baffle obstructions after atrial redirection surgery (Mustard or Senning), short segment atresia of a portion of the aorta and acquired occlusion of a portion of the iliofemoral system. In other situations, the existence, size and position of the vessel distal to an occlusion may be identified by angiography in the pulmonary artery wedge position (occluded pulmonary vein), pulmonary vein wedge position [Figure 1b] (occluded pulmonary artery), an aortopulmonary collateral vessel or other vessels that provides collateral flow to the occluded segment. Visualization of the distal vessel may be less important in the setting of an acute occlusion of a shunt or vessel with a known size and course.

## ACUTE VERSUS CHRONIC OCCLUSION

Most vascular occlusions in the pediatric and congenital cardiovascular defect patient populations are caused by thrombosis, compression of the vessel by an adjacent vessel (i.e., neoaorta compressing the left pulmonary artery after Norwood procedure),[26] twisting/kinking of the vessel or, rarely, processes like fibrosing mediastinitis. If the acutely occluded vessel is not recanalized relatively quickly, there is organization and tissue ingrowth into the occluded segment. It is generally much easier to recanalize vessels before this tissue organization proceeds to a significant extent. If recanalization can be attempted within days of the occlusion, there is an excellent probability that the occlusion can be crossed with simple guidewire manipulation. We and others have seen this to be true in pulmonary arteries, aortopulmonary shunts, iliofemoral vessels and catheter-related vascular occlusions. Successful crossing of an occlusion can be expected in over 90% of the lesions present for less than a few days if an adequate angle of approach can be achieved.

Recent work utilizing three-dimensional micro-computed tomography in an animal model beautifully shows that chronically occluded vascular segments have multiple tiny channels in and around the occluding tissue.[27] The existence of these channels may explain why small, “slippery” guidewires can sometimes successfully traverse occluded segments with surprisingly little force. In many cases of chronic occlusion, however, direct penetration of the tissue is necessary. This type of direct penetration can sometimes be accomplished with a sharp instrument such as a transeptal needle or the stiff end of a guidewire. In some cases, it may not be possible to apply sufficient force in the required direction. In these situations, radiofrequency (or much less commonly laser) energy can be used to “clear a pathway" that can be traversed by a guidewire with much less applied force. The major disadvantage of this approach is that destruction of tissue (rather than penetration) may increase the chance of bleeding if the penetrating implement exits the vascular lumen.

## OPTIMIZING THE APPROACH

**Figure 2:**
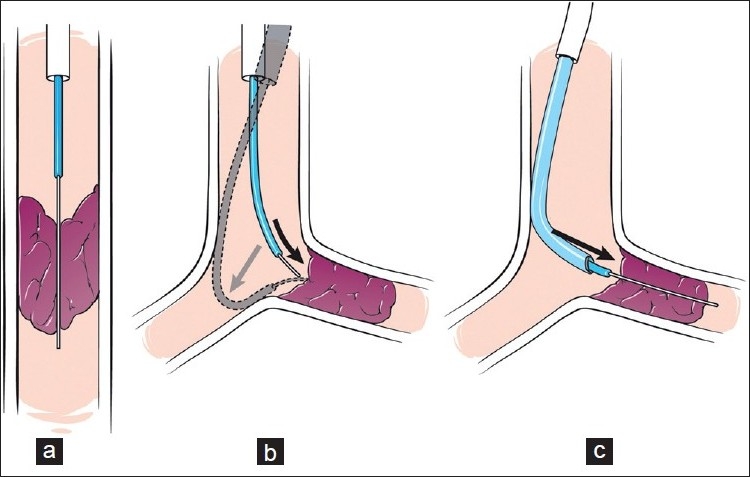
This series of diagrams illustrates the principles involved in optimizing the approach of a penetrating guidewire across vascular occlusions. A straight approach allows for the most penetrating pressure at the tip of the guidewire (a). If the occluded vessel comes off at a relatively sharp angle (i.e., the “neo” left pulmonary artery in a Glenn shunt), attempts to apply force with a guidewire through a catheter will result in the catheter being pushed away (b). This results in minimal force at the tip of the guidewire. The addition of a coaxial system (c) allows for a supporting catheter (i.e., guide catheter) to be buttressed against the vessel wall, in a stable position. This facilitates the application of enough force at the guidewire tip to cross the occluded segment

**Figure 3:**
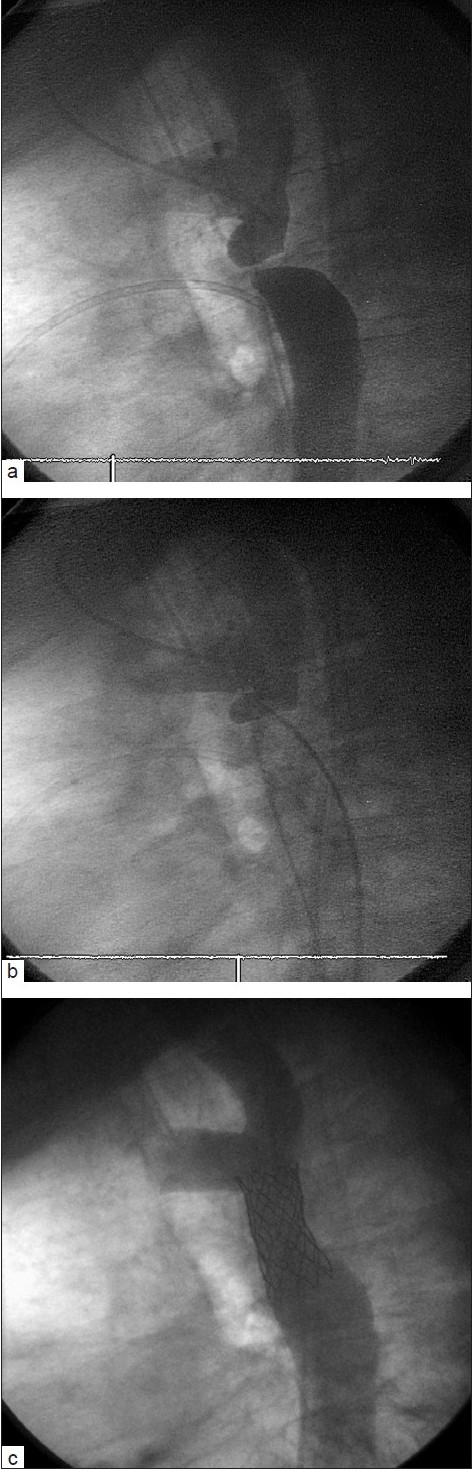
After attempts at crossing this acquired “luminal” atretic segment of the aorta with a stiff guidewire were unsuccessful, recanalization was possible with a transeptal needle, using biplane cineangiography. Simultaneous injections (a) in the ascending aorta (from an arm approach) and descending aorta were performed and used as a road map (a catheter in the LPA is seen overlying the atretic segment).The transeptal needle was advanced carefully (b) into the ascending aorta from below. A guidewire was then advanced, eventually facilitating stent placement (c)

It is always necessary to apply some degree of force to traverse an occluded vascular segment. The straightest approach possible greatly improves the transmission of force applied to the penetrating guidewire, needle or catheter by the operator. A guidewire or catheter is virtually incompressible along its length and therefore an absolutely straight course allows for the application of a great deal of penetrating pressure [Figure 2a]. If there is a bend or curve in the catheter or guidewire, however, only a portion (depending on the angle of the curve) of the force applied by the operator will be transmitted to the penetrating tip [Figure 2b and c]. The use of alternate sites for vascular access (such as the carotid or axillary artery for BT shunts) that allow for a straighter course may increase the chance of success. If the course is relatively straight, it may even be possible to use a transeptal needle as the penetrating tool if penetration with guidewires has not been successful. A transeptal needle from the jugular approach has been used by us and others to penetrate the superior Mustard baffle occlusions,[16] occluded pulmonary arteries after a Glenn procedure and in an acquired atretic aortic arch [Figure 3a, b and c].

**Figure 4:**
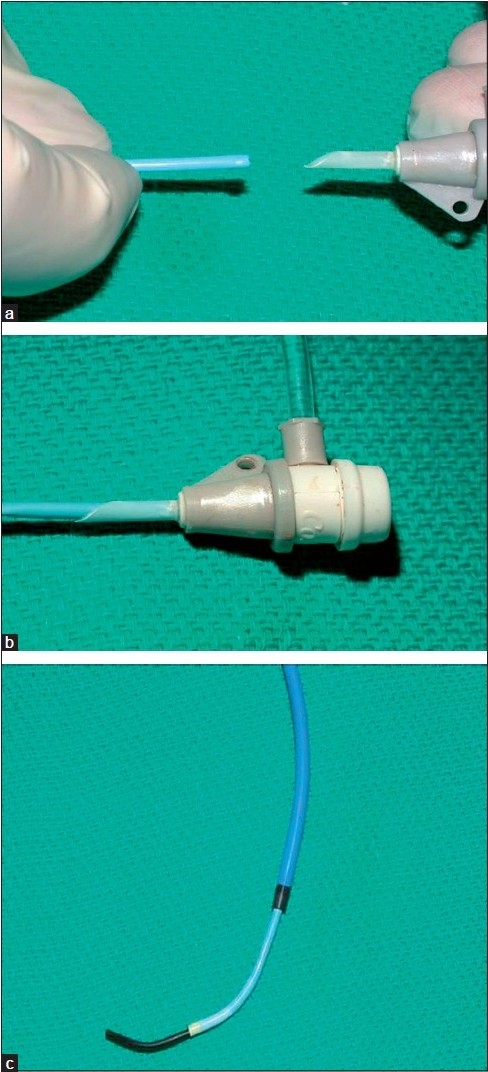
(a) A guide catheter is modified by using the hub of a sheath one Fr size less than the Fr size of the guide catheter. (b) In this instance, a 6 Fr guide catheter is cut to the appropriate length and inserted into the hub of a 5 Fr sheath (which has been cut a “bevel”). (c)The guide catheter provides a buttress for support while a hydrophilic catheter can be used with a guidewire (usually a hydrophilic guidewire) to access and cross the occluded segment. Various guide catheters can be used, depending on the angle required to access the occluded segment

In some situations, especially in the pulmonary arteries, a straight course is not possible (at least if the pulmonary artery is still connected to the right ventricle and not to a Glenn or BT shunt). If a curved course is necessary, it may be possible to use a coaxial system with a long sheath and/or a guide catheter. The guiding system needs to be buttressed along its course against the heart or other structures [Figure 2b and c]. If the guiding system can be well supported, then it may be possible to advance an inner guidewire in the necessary direction with sufficient force. We have found that a combination of a Mullins sheath, modified guide catheter, hydrophilic catheter and hydrophilic guidewire ("guide and glide" system) can be especially useful in difficult situations[28] [Figure 4a, b and c].

## AFTER THE OCCLUSION HAS BEEN TRAVERSED

After the occluding lesion has apparently been traversed by a guidewire or transeptal needle, it is essential to verify that the tip is indeed intravascular. A small angiogram through the needle or the supporting catheter may opacify the distal vessel. A guidewire should move freely in the manner expected and not appear to be confined in tissues around the intended recanalized vessel. Once the intravascular position has been confirmed, a catheter can be advanced over the guidewire and past the occlusion. If the distal tip of the guidewire can be snared, then a relatively large catheter can almost always be advanced directly over the penetrating guidewire. If there is no ability to capture the distal end of the penetrating guidewire, then we prefer to advance a small catheter into the distal vessel in most cases. A hydrophilic catheter with a lumen that matches the penetrating guidewire is often most effective. The catheter is positioned in a safe location well past the obstruction. A relatively stiff guidewire can then be advanced into this safe position.

Thrombolysis[7,9,10,13] and mechanical removal of clots with thrombectomy catheter systems[9,11,12] have been used to recanalize vessels as well, i.e. systemic to pulmonary artery shunts. However, we feel that simple guidewire techniques using balloon dilation and/or stent placement can be performed with a good result in the majority of cases. This is particularly true if the occlusion has occurred recently (within 1-2 days). While this results in distal migration of small clots, it is surprisingly rare to see clinical sequelae as a result.

In most cases, balloon dilation of the penetrating tract is performed initially with relatively small and low-profile balloons. After the tract is enlarged, a larger catheter and guidewire can be safely positioned. Simple angioplasty may be effective in some situations. In general, we try to avoid stents in small patients in areas where growth is expected. Stents may not be necessary for the treatment of acute occlusions presumed secondary to thrombosis if the probable cause for the thrombus can be eliminated. If the results of simple angioplasty appear questionable, however, we will proceed with stenting [Figure 1c and d]. Stenting almost always provides a larger lumen than angioplasty. However, the foreign material of a stent may increase the chance for subsequent acute thrombosis, and intimal ingrowth can lead to late lumen loss. We generally use aggressive anticoagulation early after placement of a stent in a completely occluded segment, especially if flow through the segment is relatively slow. We have found that it is usually safe to revert to simple antiplatelet agents in most patients if the stented segment remains patent for 2–3 months.

## PLANNING FOR THE POSSIBILITY OF COMPLICATIONS

There are very few reports of complications from attempted transcatheter recanalization procedures in the pediatric and congenital heart patients. Nonetheless, strategies for dealing with possible catastrophic vascular perforations should be considered before undertaking these types of interventions. We prefer to do these procedures with patients under general anesthesia. Blood and blood products should be immediately available. Surgical backup should be readily available in case of an emergency. In critically ill patients or early postoperative occlusions, it may be advisable to have the surgical team present or on standby.

In many situations, little or no specific treatment will be needed if a perforation occurs immediately adjacent to an occlusion with only a small guidewire. If the vessel ruptures during subsequent dilation or stenting, then temporary occlusion of the site with the dilating balloon may be successful in stopping extravasation. Covered stents may be the best treatment option if available.

## CONCLUSIONS

Transcatheter recanalization of occluded vessels in pediatric patients and patients with congenital heart disease may be an underutilized treatment option in our field. In some cases, completely occluded vessels have not been treated because of marginal results and significant risks of surgical intervention coupled with lack of awareness of the newer transcatheter options. Reports of successful transcatheter treatments are increasing in this relatively unique population. We need to remain aware of advances by other specialty groups that treat vascular occlusions in different, usually much larger, patient populations. The disease processes and types of vessels needing treatment may not be identical, but some of the principles, techniques and devices may be applicable to our patients. Some of the devices currently being investigated and actively used in interventional radiology, vascular and adult cardiology laboratories include microdissecting catheters, high-frequency mechanically vibrated wires and lumen re-entry systems.[29] While acquisition and mastery of some of these may be difficult for operators in our field due to low volumes, we suspect that some of these systems may eventually be more widely embraced if they can be demonstrated to add significantly to our current armamentarium. Recognition of the potential for transcatheter recanalization of occluded vessels, even with the current techniques outlined in this paper, will hopefully lead to more widespread consideration for this approach in our unique patient population.
